# The signals of FGFs on the neurogenesis of embryonic stem cells

**DOI:** 10.1186/1423-0127-17-33

**Published:** 2010-04-29

**Authors:** Ching-Wen Chen, Chin-San Liu, Ing-Ming Chiu, Shih-Cheng Shen, Hung-Chuan Pan, Kun-Hsiung Lee, Shinn-Zong Lin, Hong-Lin Su

**Affiliations:** 1 Department of Life Sciences, National Chung-Hsing University, Taichung, Taiwan; 2 Department of Medical Research, Changhua Christian Hospital, Changhua, Taiwan; 3 Institute of Cellular and Systems Medicine, National Health Research Institutes; Miaoli, Taiwan; 4 Department of Neurosurgery, Taichung Veterans General Hospital; Taichung, Taiwan; 5 Animal Technology Institute Taiwan; Miaoli, Taiwan; 6 Center for Neuropsychiatry, China Medical University and Hospital, Taichung, Taiwan; China Medical University Beigang Hospital, Yunlin, Taiwan; Department of Immunology, China Medical University, Taichung, Taiwan; 7 Department of Physical Therapy, China Medical University, Taichung, Taiwan

## Abstract

**Background:**

Neural induction is a complex process and the detailed mechanism of FGF-induced neurogenesis remains unclear.

**Methods:**

By using a serum-free neural induction method, we showed that FGF1 dose-dependently promoted the induction of Sox1/N-cadherin/nestin triple positive cells, which represent primitive neuroblasts, from mouse embryonic stem (ES) cells.

**Results:**

We demonstrated that FGF1, FGF2, and FGF4, but not FGF8b, enhanced this neurogenesis. Especially, FGF-enhanced neurogenesis is not mediated through the rescue of the apoptosis or the enhancement of the proliferation of Sox1^+ ^cells. We further indicated that the inactivation of c-Jun N-terminal kinase-1 (JNK-1) and extracellular signal-related kinase-2 (ERK-2), but not p38 mitogen-activated protein kinase (MAPK), inhibited the neural formation through the inhibition of ES differentiation, but not through the formation of endomesodermal cells.

**Conclusions:**

These lines of evidence delineated the roles of FGF downstream signals in the early neural differentiation of ES cells.

## Background

In the early gastrula of the chicken, temporary treatment of the primitive ectoderm with Hensen's node for 5 hours steers the ectoderm to become the neural fate [1,2]. FGF was shown to be responsible for this instructive ability of node and for the maintenance of later neural instructive signals [3,4]. FGF first activates *ERNI *during early gastrulation and consequently triggers the zinc-finger transcriptional activator, *Churchill*, and its downstream target *Sip1 *in late gastrulation [4]. In Xenopus, the study of neural induction has revealed the essential role of Ras/MAPK activation for neurogenesis in uncommitted ectoderm and in dissociated animal cap cells, suggesting that the requirement of FGF signals in neural induction is conserved in chordates [5].

ES cells, which resemble epiblast cells in the blastocyst, provide an alternative approach to the study of early development in mammals [6,7]. Several one-step neural induction models have been established. Trans-retinoic acid (RA), a pro-neural inducer, enriches the neural population in a serum-containing embryoid bodies (EBs) system [8,9]. However, RA treatment has several drawbacks, including the caudalization of the neural fate, blockage of forebrain induction, and the disruption of normal embryogenesis [9-11]. Co-culture of ES cells with mouse skull-derived stromal cells, such as PA6 cells, or bone marrow-derived cells, such as MS5 cells, efficiently induces the ES cells to become neuron lineages [8,12]. However, the factors contributing to this stromal-derived inducing activity are still uncharacterized. ES cells cultured in serum-free Neurobasal medium with N2B27 supplement efficiently differentiate into Sox1^+ ^neural precursors, which represent the earliest committed neuroblast cells in the developing embryo [13,14]. Specific neuronal subtypes, such as dopaminergic and serotoninergic neurons, are derived from the Sox1 neuroblasts by the addition of defined patterning factors. Although the Neurobasal/N2B27 model provides a simple monoculture differentiation system for ES cells, these cells often undergo apoptosis on days 3 to 5. Recently, an efficient neural-induction monoculture system with a high survival rate for differentiating ES cells was developed and termed as serum-free embryoid bodies formation (SFEB) method [15]. This simple and reproducible system consists of defined components and is suitable for the exploration of downstream FGF signals in the early neurogenesis of mammals.

## Methods

### Cell culture and differentiation

Sox1-GFP knock-in ES cells (46C), from Dr. Austin Smith (University of Cambridge, UK), and ESC 26 cells, were both well-characterized and germline transmissible [14,16]. The culture condition of both cells [14,16] and the SFEB method [15] has been described previously in detail.

### Reagents

Human recombinant FGF2, FGF4 and FGF8b were all from R&D Systems. Recombinant human FGF1 was prepared from Prof. Chiu in Institute of Cell and Systems Medicine, the National Health Research Institutes, Taiwan [17]. Synthetic inhibitors of FGF signaling, including SU5402, LY294002, SB203580, and SP600125, were from Calbiochem; U0126 was purchased from Tocris.

### Stable cell establishment

The plasmid Flag-DsRedT4-NLS was a gift from Tim Shroeder at Helmholtz Center Munich, Institute of Stem Cell Research, Germany. The genes of JNK dominant negative mutants, Flag-JNK1a1apf and Flag-JNK2a2apf [18,19], were obtained from Addgene http://www.addgene.org and fused with a IRES-DsRed as a reporter. The plasmids were transfected into ES cells with lipofectamine 2000 (Invitrogen). After selection with 0.4 mg/ml G418 for two weeks, stable clones with red fluorescence were picked up and maintained with 0.2 mg/ml G418. The selected ES cells showed normal ES cell morphology and pluripotent gene expression (data not shown).

### Immunocytochemistry

Cells were fixed in 4% cold paraformaldehyde and permeabilized with 0.3% Triton-X 100. Immunocytochemistry was performed with the following primary antibodies: OCT3/4 (1:500, Santa Cruz), Nanog (1:100, Cosmo Bio, Japan), Sox2 (1:4000, Chemicon), N-cadherin (1:100, DSHB, Iowa), FGF receptor 1 (FGFR1) and FGFR3 (both 1:100, Santa Cruz), FGFR2 (1:500, Abcam) and GFP (1:1000, Aves Labs). Images of immunostaining were captured usinga fluorescent microscope (Nikon ECLIPSE 80I) or confocal microscope (LSM510 Meta, Zeiss).

### Flow cytometry

Sox1-GFP ES cells were fully dissociated and analyzed with flow cytometry (FC500, Beckman Coulter). Apoptosis was measured by staining for Annexin V (AbD Serotec) at room temperature for 10 min in the dark.

### RT-PCR analysis

Total RNA was isolated from ES cells using REzol™ C&T reagent (Protech technology, Taiwan). Primers were applied to detect the expression of FGFR1 (5'-CAC ACT GCC TTC TCC TCC TC-3', 5'-CTC TGC CTC CCT GTC TTC TG-3'), FGFR2 (5'-GGG GAT GTG GAG TTT GTC TG-3', 5'-GCT TCT TGG TCG TGG TCT TC-3'), FGFR3 (5'-CGG CTA CCT GTG AAG TGG AT-3', 5'-GCT TGG TCT GTG GGA CTG TT-3'), FGFR4 (5'-AGG AAA TGT GGC TGC TCT TG-3', 5'-GGT GTG TCC AGT AGG GTG CT-3'), Sox1 (5'-CCT CGG ATC TCT GGT CAA GT-3', 5'-TAC AGA GCC GGC AGT CAT AC-3'), and G3PDH (5'-GTG AAG GTC GGT GTG AAC G-3', 5'-GGT GAA GAC ACC AGT AGA CAC TC-3').

### Western blot analysis

ES cells were lysed in RIPA buffer (50 mM Tris pH7.5, 150 mM NaCl, 10 mM EDTA, 1% NP-40, 0.1% SDS) plus a cocktail of proteinase inhibitors (Sigma-Aldrich). Denatured proteins were separated by 10% SDS-PAGE and then transferred to PVDF membranes. Samples were detected with antibodies to ERK1/2, phosphoERK1/2 (pERK1/2), p38 and pp38, JNKs and pJNKs, AKT and pAKT. All MAPK-related antibodies were from Cell Signals and diluted 1:1000 for immunoblotting. Chemiluminescence of immunoreactive bands was detected using secondary horseradish peroxidase-conjugated antibodies (Jackson ImmunoResearch) and ECL reagents (Amersham).

## Results

### FGF1 enhanced the generation of Sox1^+ ^cells from ES cells

**Figure 1:**
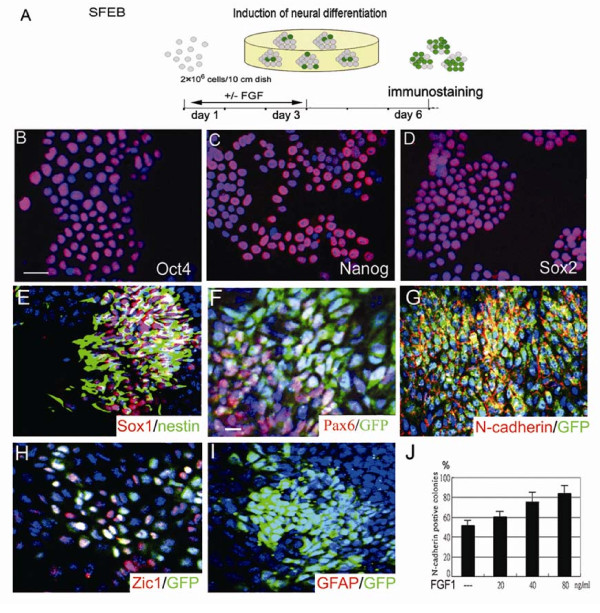
**The characteristics of the ES cells and their neural derivatives**. (A) Schematic procedure of SFEB for neural induction of ES cells. Undifferentiated ESC 26 cells were characterized by pluripotent markers such as Oct4 (B), Nanog (C) and Sox2 (D). The 46C ES-derived GFP^+ ^cells were coexpressed with neural markers, such as nestin (E), pax6 (F), N-cadherin (G), Zic1 (H), but not GFAP (I) on day 6. Nuclei of ES cells were stained with DAPI in blue (B-I). ESC 26 cells were treated with 20, 40, and 80 ng/ml FGF1 from day 1 through day 3 and the N-cadherin^+ ^colonies were estimated under fluorescent microscope (J) on day 6 from three independent experiments. A cell cluster with over 50 μm was counted as a colony and a colony was N-cadherin positive if over half of the cells in the colony expressed N-cadherin. Scale bar, 10 μm in B.

**Figure 2:**
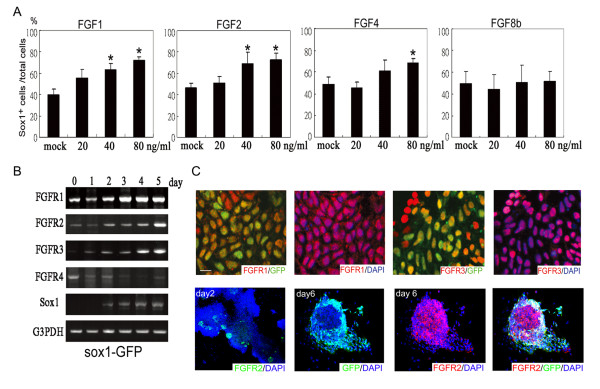
**The FGF effects on the neurogenesis of ES cells and the FGFR expressions in ES cells**. (A) After treatment with FGF1, FGF2, FGF4, and FGF8b from day 1 to day 3 using the SFEB method, the numbers of 46C ES-derived Sox1-GFP^+ ^cells were estimated by flow cytometry on day 6 (n = 3 for each panel). (B) On indicated days, FGFRs in 46C ES cells were analyzed by RT-PCR. (C) Expression of FGFRs and the GFP^+ ^ES cells was analyzed by immunostaining on day 6 or day 2. Single GFP positive cells were indicated by arrow. Nuclei of all cells are revealed by DAPI staining in blue. Scale bar, 10 μm in C. *, p < 0.01, Anova test.

Two germline-transmissible mouse ES cell lines, ESC 26 and Sox1-GFP knock-in cells (46C), were used in this study and the ESC 26 cell was characterized with the expression of pluripotent makers (Fig. 1B to 1D). After dissociation, ES cells were cultured at 2 × 10^6 ^cells/10 ml in a defined, serum-free, neural differentiation medium (SFEB method) (Fig. 1A), which is an efficient neural induction method with rare mesendoderm formation [15]. We showed that ES-derived Sox1-GFP^+ ^cell was coexpressed several neural markers, such as nestin, pax6, N-cadherin and Zic1 (Fig. 1E to 1H). In addition, GFAP was not detected in differentiating 46C cells on day 6 (Fig. 1I), indicating that the Sox1^+ ^cells under the SFEB culture represented primitive neuroblast cells [15]. Exogenous FGF1, applied from day 1 through day 3, dramatically enhanced the neural induction of ESC26 and 46C cells in a dose-dependent manner, as revealed by the counting of N-cadherin^+ ^colonies (Fig. 1J) and FACS analysis on day 6, respectively (Fig. 2A). These results suggest that FGF was sufficient to promote the formation of neuroblast cells derived from ES cells.

We next tested the effects of different FGFs on neural formation of ES cells. FGF1, FGF2, and FGF4 all showed significantly elevated neural induction in 46C cells (Fig. 2A). However, FGF8b, even at the high concentration of 80 ng/ml, failed to enhance the neural induction of ES cells (Fig. 2A). We further investigated the expression of FGFRs in ES cells during neural induction and found that the expression of FGFR4 gradually declined (Fig. 2B), which is in agreement with the finding that FGFR4 is excluded from the neuroectoderm of mouse embryos [20]. In contrast, FGFR1, FGFR2, and FGFR3 expressions were significantly increased during the conversion of ES into neuroblast cells. Immunocytostaining revealed that both FGFR1 and FGFR3 were detected in cytosol and nuclei in neural derivatives (Fig. 2C). On day 6, GFP^+ ^signals were colocalized with FGFR1- and FGFR3-expressing cells, suggesting that both signals may be involved in neurogenesis (Fig. 2C). RT-PCR and immunostaining, shown in Figs. 2B and 2C, indicated that the expression of FGFR2 in differentiating ES cells was robustly induced and was localized on the cell membrane and cytosol, rather than in the nucleus. We also found that FGFR2 was not completely coexpressed with the GFP in 46C cells on day 6 (Fig. 2C), suggesting that FGFR2 is involved in the formation of subtypes of neurons. Taken together, these results suggest that FGFR1 and FGFR3 are generally required for neural induction and FGF8b is incompetent on the enhancement of neurogenesis of ES cells.

### Neural induction enhanced by FGF was not mediated through the anti-apoptosis or cell proliferation on Sox1^+ ^cells

**Figure 3:**
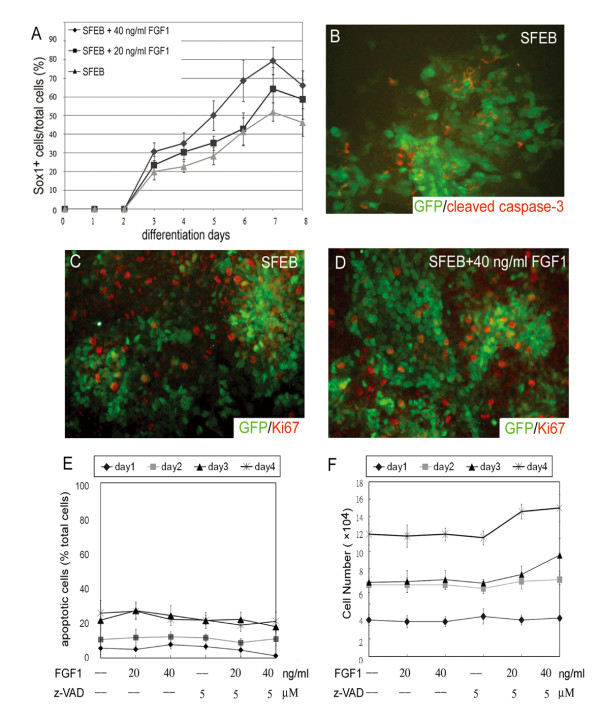
**The apoptosis and the proliferation on committed neuroblast cells**. (A) The induction of Sox1-GFP^+ ^cells from 46C cells were detected by flow cytometry under the SFEB and SFEB/FGF1 condition. (B) The differentiating ES cells were labeled with cleaved caspase-3 (red), which detects the cleaved fragment of caspase-3 (17/19 kDa), in Sox1/GFP^+ ^cells on differentiating day 4. (C, D) Proliferating GFP^+ ^cells were marked with the nuclear staining of ki67 on day 4. (E) Total apoptotic cells, characterized with Annexin-V labeling, were estimated by flow cytometry after FGF and/or z-VAD-fmk, a membrane-permeable pan-caspase inhibitor, from day 1 to day 4. Culture media were changed every day. (F) Total cell numbers were counted in triplicate using trypan blue exclusion at indicated times.

We treated 46C ES cells with or without FGF1 from day 1 through day 3 and detect the Sox1-GFP^+ ^cells from day 1 to day 8 (Fig. 3A). The number of Sox1^+ ^cells became 20% of total cells on day 3 and reached the plateau, 50% of total cells, on day 7. Treatment of FGF1 consistently and dose-dependently enhanced the neurogenesis on day 3 through day 7. We also found that FGF treatment can promote but cannot shorten the time of the neural induction from ES cells. The Sox1-GFP^+ ^cells did not appear on differentiation day 2, regardless of the FGF1 treatment.

The increase of Sox1^+ ^cells in the FGF1-treated condition may result from enhanced proliferation and/or reduced apoptosis of neuroblast cells. To test these possibilities, FGF1 was incubated with the 46C cells, and the apoptosis and proliferation of Sox1^+ ^cells were analyzed by staining of activated caspase-3 and Ki67, respectively. Double staining of cleaved caspase-3 and GFP revealed that less than 5% double positive cells were detected (Fig. 3B). Similar results were obtained in FGF1-treated Sox1^+ ^cells (data not shown). The percentages of Ki67^+ ^cells in Sox1^+ ^population were 24.75% (196/792) and 25.48% (362/1421) in SFEB- and SFEB/FGF1-treated cells respectively (Fig. 3C and 3D), demonstrating that FGF-triggered neurogenesis may not mediated through the enhancement of Sox1 cell proliferation.

We also found that on day 1 through day 4, the total number of apoptotic cells was not reduced after treatment with 40 ng/ml FGF1, or with 5 μM of a pan-caspase inhibitor, z-VAD-fmk. Even after the addition of both FGF1 and z-VAD-fmk, the rescue of apoptotic cells was not significant (Fig. 3E). The total ES cell population was also counted on differentiation days 1 to 4. No statistical significance in number was seen after treatment with FGF1 and/or z-VAD-fmk (Fig. 3F). In sum, these results suggest that the FGF-steering neurogenesis mainly depends on the enforcing differentiation of ES cells, rather than on anti-apoptosis or cell proliferation.

### Neural induction of ES cells was mediated through the activation of MAPK pathways

**Figure 4:**
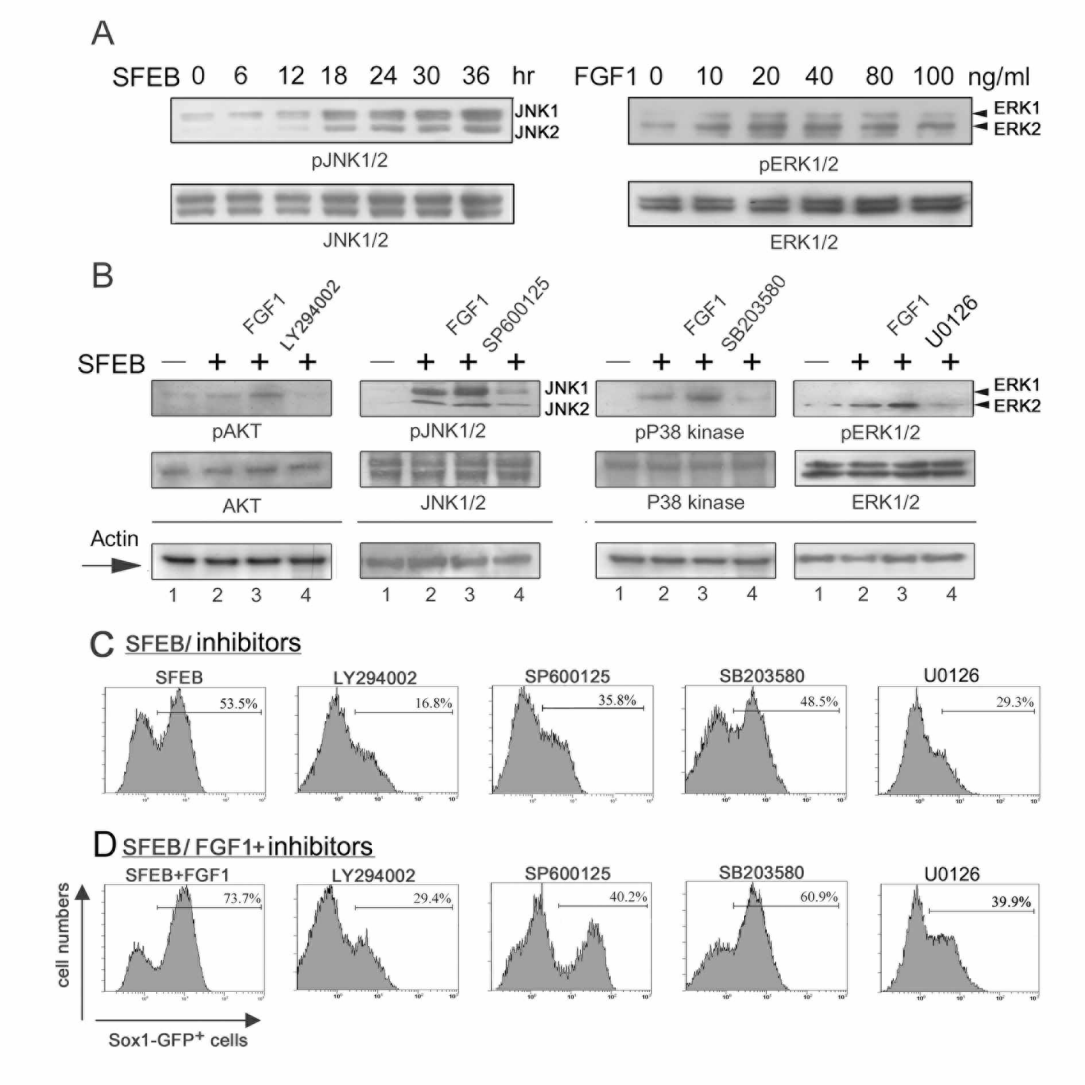
**Effects of MAPK inhibitors on neural induction of ES cells**. (A) Total cell lysates were collected from differentiating ES cells at indicated times under SFEB condition. Kinetic JNKs activation was analyzed by western blot. FGF1 dose-effect on differentiating ES cells was revealed by ERK phosphorylation at 30 min differentiation. (B) Downstream FGF signals were further detected with individual specific antibodies at 12 hr post-treatment of 40 ng/ml FGF1 (lane 3), or with inhibitors (lane 4) of PI3K/AKT (LY 294002, 10 μM), JNK1/2 (SP 600125, 10 μM), p38 MAPK (SB 203580, 20 μM), and ERK1/2 (U0126, 5 μM). After treatment with the inhibitors (C) or FGF1 (40 ng/ml) plus the inhibitors (D) from day 1 to day 3, the derived cells were collected for FACS analysis on day 6. The same concentrations of reagents were applied in these experiments. Representative results were shown from experiments done at least in triplicate.

Given that phosphorylated intracellular domains of FGFRs activate downstream phosphoinositide-3 kinase (PI3K)/AKT and three major serine/threonine MAPKs, including ERK 1/2, JNKs, and p38 kinases, we further investigated which MAPK pathways were responsible for the FGF-dependent neural induction. We found that single suspended ES cells continued to initiate phosphorylated JNK during differentiation (Fig. 4A). Significant enhancement of ERK activation was observed in 20 ng/ml FGF1-treated ES cells, providing the linkage of biochemical evidences of FGF signal with its pro-neural function. FGF1 promoted the AKT phosphorylation and the activities of all three MAPKs in differentiating ES cells at 12 hr differentiation (Fig. 4B). Immunoblotting showed that the total amount of AKT, JNK, p38 MAPK, and ERK1/2 protein expression was not altered between control and SFEB conditions. Especially, JNK1 and ERK2 were the major phosphorylated isoforms of JNKs and ERKs in the differentiating ES cells, respectively.

Specific pharmacological inhibitors of MAPKs, shown affecting their respective kinase targets in Fig. 4B, were administrated to delineate the kinases involved in neurogenesis. We found that a PI3K/AKT inhibitor, LY294002, significantly reduced the formation of Sox1-GFP^+ ^cells under SFEB and SFEB/FGF1 conditions (Fig. 4C and 4D).

**Figure 5:**
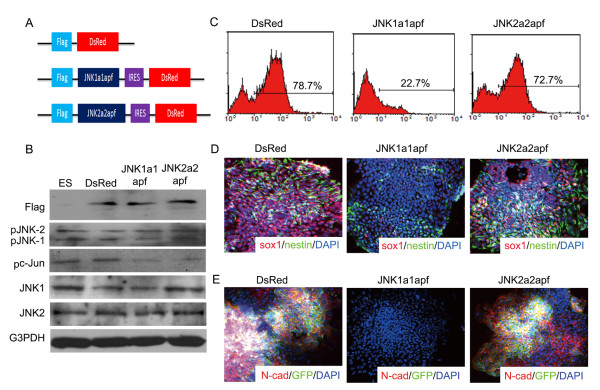
**Genetic inhibition of JNKs in differentiating ES cells**. (A) Flag-tagged dominant-negative mutants of JNK1 and JNK2 (JNK1a1-apf and JNK2a2-apf) were conjugated with IRES-DsRed for the tracing of the consistently expressing cells. (B) The expression of flag, phosphorylated JNKs, phosphorylated c-Jun (pc-Jun) and total amount of JNK1 and JNK2 were revealed by western blot. (C) Their efficiencies of neural formation were estimated by FACS analyses. The expressions of neural markers are also examined, such as Sox1 (D), nestin (D) and N-cadherin (N-cad) (E).

Intriguingly, a JNK inhibitor and an ERK inhibitor, SP600125 and U0126, respectively, dramatically blocked the neural formation of ES cells and abolished the FGF-mediated neurogenesis (Fig. 4C and 4D). Nevertheless, there was no significant reduction of Sox1-GFP^+ ^cells after treatment with p38 kinase inhibitor, in both exogenous FGF present or absent condition (Fig. 4C and 4D). In addition, to verify the role of JNK isotypes in neural differentiation of ES cells, stable clones expressing the JNK1 and JNK2 dominant negative mutants (JNK1a1apf and JNK2a2apf) were established (Fig. 5A and 5B). We found that specific inhibition of JNK1, but not JNK2, significantly reduced the formation of Sox1^+ ^and N-cadherin^+ ^cells (Fig. 5C, 5D and 5E), indicating that JNK1 is essential for the neural induction of ES cells.

### Response-time windows for the FGF-mediated neurogenesis

**Figure 6:**
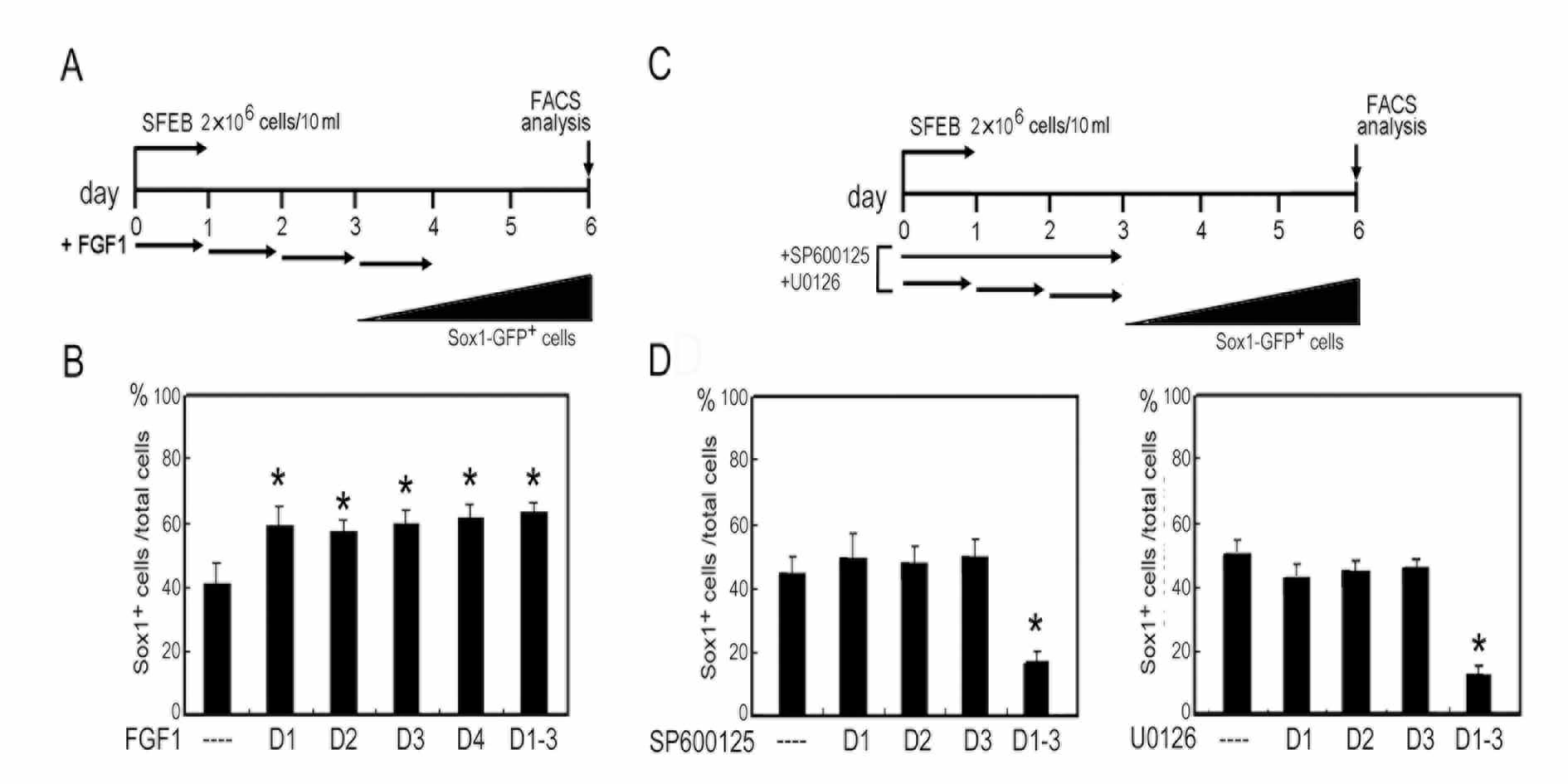
**Response windows of FGF and MAPK inhibitors in differentiating ES cells**. (A) FGF1 at 40 ng/ml was applied to 46C ES cells on individual days (D1, D2, D3, D4) or from day 1 through 4 (D1-4). (B) Derived GFP^+ ^cells were analyzed by FACS on day 6. Independent experiments done in triplicate are illustrated. (C) As the indicated experimental conditions, the induction of Sox1-GFP^+ ^cells on day 6 was shown in (D) after FACS analysis. SP600125 and U0126, 10 μM and 5 μM, respectively.

To verify the FGF response windows during ES differentiation, 40 ng/ml FGF1 was incubated with 46C cells for 24 hr on individual day 1 to 4 (Fig. 6A). ES-derived neural cells were analyzed on day 6 by FACS. FGF1 treatment in the first 24 hr window was sufficient to promote Sox1 cell induction (Fig. 6B, the lane D1). Neurogenic effects were also observed when the ES cells were incubated with FGF1 on day 2 or 3 (Fig. 6B, the lane D2 and D3). This result argues that transient FGF activation is sufficient to enforce early cell-fate commitment and neural induction of ES cells. In contrast, JNK and ERK inhibitors caused only a short-term reduction of neurogenesis and a delay in commitment. As shown in Figs. 6C and 6D, neural inhibition was observed on day 6 when MAPK signals were constantly depressed throughout days 1 to 3 (Fig. 6D; the lane D1-3). Transient treatments of both inhibitors on individual days did not show the suppression of neural induction (Fig. 6D; the lane D1, D2 and D3). Interestingly, we also found that GFP^+ ^cell population with the treatment of MAPK inhibitors throughout days 1 to 3 gradually increased from 26 ± 5.5% on day 6 to 55 ± 6.7% of total cells on day 9 (data not shown), suggesting that inhibition of JNK and ERK retards the ES cell commitment, rather than promotes non-neural lineages.

### Cell lineages of the ES cells treated with MAPK inhibitors

**Figure 7:**
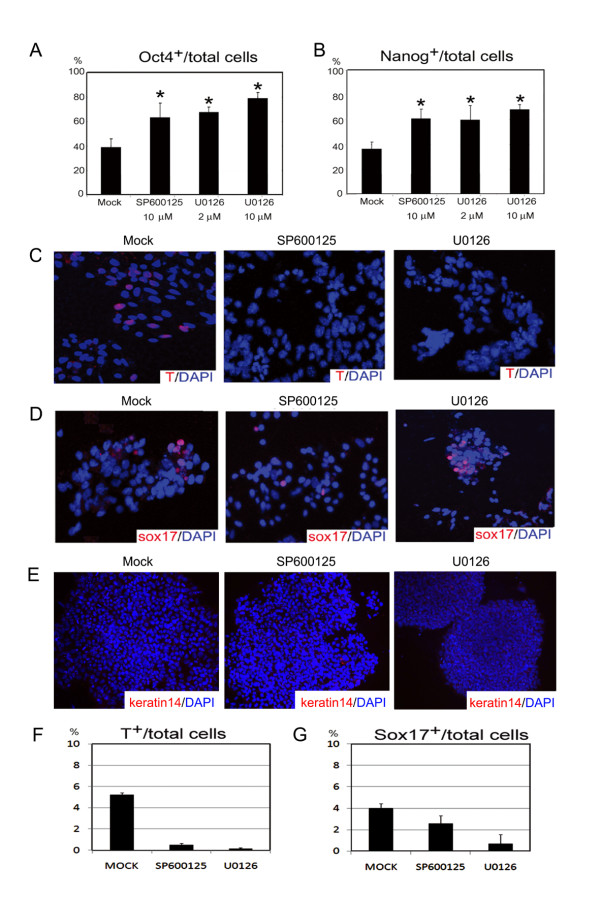
**Both inhibitors of JNK and ERK retarded ES differentiation**. After treatment with 10 μM SP600125, 2 or 10 μM U0126 from days 1-3, ES cells were plated on 0.1% matrigel-coated glasses and stained with anti-Oct4 (A) and anti-Nanog antibodies (B) on day 6. The ratio of undifferentiated pluripotent ES cells to total DAPI^+ ^cells (n>500 cells) was estimated from experiments done in triplicate. Brachyury (T) (C), Sox17 (D) and cytokeratin 14 (E) expressions, representing mesodermal, endodermal and surface ectodermal cell lineages respectively, were examined in ES cells on day 6 with SFEB treatment. Nuclei of all cells are seen by DAPI staining in blue. The statistic results of the cell numbers in panel C and D were also estimated, respectively (E, F).

Reduction of the neural induction by the JNK and ERK inhibitors could be caused by the increased undifferentiating ES cells or non-neural lineages. In this study, we demonstrated that inactivation of both JNK and ERK enhanced the expression of pluripotent markers Oct4 and Nanog in differentiating ES cells on day 6 (Figs. 7A and 7B), indicating that both phosphorylated JNK and ERK are negative regulators of self-renewal of ES cells. It is recently documented that ERK2 null ES cells fail to commit into neural and mesodermal cells [21-24]. Similarly, rare brachyury (T) expressed cells were found in SP600125- and U0126-treated ES cells, compared to 5.2 ± 0.2% brachyury-positive cells in the total population under SFEB (Fig. 7C and 7E). The Sox17^+ ^cells, representing endoderm of differentiating ES cells, only showed less 5% of total ES cells on day 6 under the SFEB condition (Fig. 7D). No significant elevation of Sox17^+ ^cells was observed in JNK/ERK inhibitors treated ES cells (Fig. 7F). In addition, we also did not find the appearance of cytokeratin 14 (K14) positive cells, representing the epidermal precursor cells, in the SFEB-differentiating ES cells even after the treatment of MAPK inhibitors. These results demonstrated that the reduction of neural formation by the inactivation of MAPK was caused by the blockage of ES differentiation, rather than by the enhancement of formation of mesoendodermal nor epidermal lineages.

## Discussion

Neural induction requires sequential signals to direct uncommitted ectoderm into the definitive neural plate [25]. Cumulative evidence supports the fact that FGF is an essential factor for neurogenesis [26,27]. Interestingly, activation of the Ras/MAPK pathway, rather than the diluted BMP ligands, has been shown to be responsible for the neural cell fate of the fully dissociated animal cap cells, arguing against the simplistic neural default model [5]. The primitive streak- or organizer-derived BMP inhibitors are not the only signals required for neurogenesis. FGF and the other developmental cues, such as Wnt and Notch, also participate in neural induction in a sophisticated manner [25].

It is noteworthy to emphasize that the activation of MAPK during ES differentiation may not solely depend on FGFR signals and other neural instructing factors could also contribute to the neural induction through JNK or ERK activation, such as insulin-like growth factor (IGF) [28]. Treatment of JNK and ERK inhibitors should simultaneously abolish the endogenous receptor tyrosine kinase signals of differentiating ES cells. Here we showed that neural induction of ES cells was accompanied with the elevated expression of FGFRs and the activation of MAPK pathway (Figs. 2B, 4A and 4B). Pharmacological evidences (Fig. 4C) further supported that differentiation into primitive neuroepithelial cells relied on the activation of both JNK and ERK pathways, but not the p38 MAPK pathway (Fig. 4C). Exogenous FGF-triggered neurogenesis was completely reduced by the JNK and ERK inhibitors (Fig. 4D). Taken together, these data highlights the importance of FGFR activation and of individual MAPK signals in the ES-neuron conversion.

Both pharmacological and genetic evidences support the important role of JNK1 for the neural induction of ES cells (Fig. 4C, D and 5). These results are consistent with the previous finding that JNK1^-/- ^ES cell has a significant reduction in RA-triggered neurogenesis and that JNK/Stress-associated activated protein 1 (JSAP1) is involved in early embryonic neurogenesis [29,30]. While a neural tube defect is only observed in JNK1/JNK2 double-knockout mice and a JNK1 and JNK2 single-null embryo is normal [31]. It is important to further explore the reason of discrepancy between in vitro and in vivo data and the JNK regulatory networks which participate in neural fate decision and the development of primitive neuroectoderm.

Genetic manipulation has shown that ERK1-null mice are healthy after birth, whereas disruption of the ERK2 gene results in abnormal trophectodermal and mesodermal development [32,33]. In vitro ES differentiation has also revealed that inhibition of ERK2 completely blocks neural and mesodermal formation, suggesting that ERK2 is essential for the initiation of cell fate commitment of epiblast cells [21,24]. In this study, we showed that inhibition of MAPK signals sustained the undifferentiated status and the expression of pluripotent markers under the SFEB condition. In future studies, it will be important to understand how the regulatory networks of MAPKs are affected after deprivation of LIF and how they initiate somatic cell induction in ES cells.

## Conclusions

Based on a simple and efficient neural induction method, we demonstrate that FGF-triggered neurogenesis of ES cells is not involved in cell proliferation or inhibition of apoptosis. Activation of the ERK2 and JNK1 pathways, rather than p38 MAP kinase, is mainly responsible for the neural induction of ES cells. Release of pharmacological inhibition re-initiated the ES differentiation and neurogenesis, indicating that the FGF pathway participates in the initiation of ES commitment into embryonic cell lineages.

## List of abbreviations

ESC: embryonic stem cell; FGF: fibroblast growth factor; MAPK: mitogen-activated protein kinase; SFEB: serum-free embryoid body-like formation.

## Competing interests

The authors declare that they have no competing interests.

## Authors' contributions

CWC, SCS, HCP and HLS carried out the neural differentiation and drafted the manuscript. KHL provided the mES cells and participated in the design of the study. CSL, IMC SZL and HLS participated in the design of the study and performed the statistical analysis. All authors read and approved the final manuscript.
